# Neuroendocrine Tumor Therapy with Lutetium-177: A Literature Review

**DOI:** 10.7759/cureus.3986

**Published:** 2019-01-30

**Authors:** Muhammad Haisum Maqsood, Asim Tameez Ud Din, Ameer H Khan

**Affiliations:** 1 Epidemiology and Public Health, King Edward Medical University / Mayo Hospital, Lahore, PAK; 2 Internal Medicine, Rawalpindi Medical College, Rawalpindi, PAK; 3 Internal Medicine, Allama Iqbal Medical College, Lahore, PAK

**Keywords:** lutetium, neuroendocrine tumors, gastroenteropancreatic tumors

## Abstract

The worldwide incidence of neuroendocrine tumors (NETs) has been increasing. They are a very diverse group of tumors which are commonly found in the gastrointestinal and bronchopulmonary tracts. These tumors usually express somatostatin receptors. Therefore, somatostatin analogs are used for symptom relief as well as treatment. Of the many therapeutic options available, peptide receptor radionuclide therapy (PRRT) has been shown to be very promising. In January 2018, the Food Drug and Authority (FDA) approved ^177^Lu-Dotatate for use in gastroenteropancreatic neuroendocrine tumors (GEP-NETs). Lutetium is a lower energy beta-emitting radionuclide. The therapeutic use of lutetium-^177^ (^177^Lu) has shown better results in advanced gastroenteropancreatic and bronchial neuroendocrine tumors when compared with other therapies available. Adverse effects associated with this therapy include myelotoxicity and nephrotoxicity as the radiopeptides are reabsorbed and accumulate in the renal interstitium. Everolimus is a good and safe option in patients pretreated with ^177^Lu-Dotatate. Lutetium, in combination with somatostatin analogs, has proven efficacy to treat gastroenteropancreatic neuroendocrine tumors in candidates with somatostatin receptor-positive advanced tumors and normal renal function. This therapy has great potential as it decreases tumor size, improves symptoms, and improves quality of life.

## Introduction and background

The worldwide incidence of neuroendocrine tumors (NETs) has been increasing [1]. This can be explained by an improvement in imaging techniques and diagnosis. NETs are very diverse and can be divided on the basis of their primary site, histologic grade, and genetic makeup. The growth rate of gastroenteropancreatic neuroendocrine tumors (GEP-NETs) is very slow [2]. Since there is great diversity in these tumors, treatment strategies should also be tailored to particular types because many treatment options are now available [3]. Of the available treatment options, radiolabelled somatostatin analogs (SSAs) are the only ones with a well-defined biomarker, which is the expression of the somatostatin receptors (SSTR) [4].

Neuroendocrine tumors can originate from the gastrointestinal tract and the bronchopulmonary tract. They are also broadly classified as functional and non-functional tumors based on the presence or absence of specific symptoms. Functional tumors manifest symptoms by producing bioactive chemicals. Nonfunctioning tumors do not produce active substances and usually present as widespread metastatic disease. SSAs are commonly used not only for symptom control but also for decreasing the tumor growth and improving the quality of life in affected individuals [5-7]. Carcinoid tumors, based on their origin, can be further divided into three groups, which are foregut, midgut, and hindgut [8]. The most common foregut-derived tumors are of bronchial and gastric origin [9]. Presence of somatostatin receptor Type 2 can be detected in such tumors with Indium-111 (^111^In)-octreotide scintigraphy [10] and radiolabelled somatostatin analogs can be used for therapy.

NETs have the ability to synthesize, store, and secrete neuroamines and peptides [11]. The carcinoid syndrome, characterized by flushing, diarrhea, and right-sided valvular heart disease, is usually caused by a midgut metastasized NET [12]. Patients with localized NETs are treated with surgery, but 40% of patients already have metastasized disease at diagnosis and require systemic treatment [13]. Targeted therapy has been utilized to treat these tumors, which includes somatostatin analogs (SSAs) and peptide receptor radionuclide therapy (PRRT), as these tumors express SSTRs. Low and intermediate grade tumors express these receptors at a higher density as compared to high-grade tumors [14].

In January 2018, the Food Drug and Authority approved ^177^Lu-Dotatate for use in GEP-NETs [15]. This literature review will highlight the clinical features of using lutetium-177 (^177^Lu)-based PRRT in these tumors.

## Review

Mechanism of action and use

Radiolabelled SSAs bind SSTRs on tumor cells and are internalized and later stored in lysosomes, thereby delivering the radioisotope to the tumor cells [16]. This is how the tumor cells are targeted in this therapeutic technique [17]. ^177^Lu is a β-emitter and has a higher range and energy as compared to other radionuclides. Variation in the tumor absorbed fraction for lutetium was less in the models studied as compared to the other radionuclides [18]. Its emission of γ-rays also makes it useful for monitoring tumor response [19]. Radionuclides other than ^177^Lu, such as yttrium-90 (^90^Y) and ^111^In, have also been used in PRRT.

Patients with somatostatin receptor (SSTR)-positive NETs and near-normal kidney and bone marrow function are good candidates for PRRT. ^177^Lu-Dotatate, the most commonly used radiopeptide, has been shown to have comparable efficacy and a better hematological toxicity profile than yttrium-90 Dotatoc (^90^Y-Dotatoc) [20-21]. In many studies, ^177^Lu-Dotatate has been shown to have a good response rate and a positive impact on the quality of life [22]. ^177^Lu-Dotatate, in comparison with high-dose octreotide, has been shown to result in a 79% reduction in risk of progression or death [23]. Retreatment with the same or a different radiopeptide has been shown to be safe but less effective than the initial treatment. Radiopeptides have been tried sequentially or in combination with other drugs. Different radiopeptides have also been used in combination with success but definitive proof requires prospective randomized trials. PRRT has proven efficacy as a neoadjuvant treatment for NETs [24]. Its combination with other drugs needs further research.

In addition to SSRs, mutated epithelial cadherins (E-Cad) are also exclusively found in gastric cancer cells, which makes them a preferable target for therapy using immunoglobulins [25]. Antibodies against the mutated delta 9 E-cadherin (d9 E-Cad) are combined with bismuth-213 (^213^Bi), which is an α-emitter [26]. The α-particles cause necrosis in the cancer cells [27], whereas lutetium, as discussed, is a β-emitter and these particles have a 50x greater range as compared to α-particles. Thus, where α-emitter therapy is useful for early-stage disease, β-emitter therapy has been explored for comparatively more disseminated disease.

It has been demonstrated that ^177^Lu peptide receptor radionuclides are effective in treating patients with metastasized neuroendocrine tumors [28]. The therapy increased the global health of the treated patients, especially those that had a proven tumor regression. The treatment improved not only the functional status but also the symptoms experienced by the patients [29].

Side effects

PRRT can cause myelosuppression by irradiating the bone marrow, even though it is mild and reversible. Ten percent of patients treated with Lu-Dotatate develop World Health Organization (WHO) Grade 3/4 hematotoxicity [30]. Radiopeptides can accumulate in the renal interstitium because of their reabsorption in the proximal tubule and cause damage. This can be reduced by administering a positively charged amino acid infusion [31]. Other less common adverse effects include lymphopenia, acute leukemia, and myelodysplastic syndromes.

A dose-limiting factor for the use of ^177^Lu-PRRT is myelotoxicity. A method to mitigate the myelotoxicity, such as extracorporeal affinity adsorption treatment (ECAT), can be used [31]. It can decrease the blood content of ^177^Lu after treatment is given and thus reduce the myelotoxicity. Another method of increasing the clearance of the radionuclides is modifying the conjugates with carbohydrates [32].

Brabander et al. studied the side-effects of this therapy related to bone marrow and kidney function. Giving an infusion of lysine and arginine before therapy has resolved the nephrotoxicity. ^177^Lu-octreotate is also implicated in causing cytopenias and myelodysplastic syndrome but this is relatively uncommon. An increase in aminotransferases (aspartate transaminase and/or alanine transaminase) was observed in a few cases. Acute leukemia (AL) occurred during follow-up in 0.7% of patients [33].

Combination therapies

Radiopeptides are used for targeting tumors for localized or internal radiotherapy [34]. In the case of GEP-NETs, somatostatin analogs, combined with radionuclides, are used as these tumors expresses SSTRs. As there are many types of SSTRs expressed by tumors, a radiopeptide that can bind with most of these receptors is desirable. A new radiopeptide based on somatostatin was developed called DOTA-(Nal3)-octreotide (DOTA-NOC). This ligand can target more SSTRs and possibly treat a larger spectrum of tumors. DOTA-NOC can be used with radionuclides, such as ^90^Y, ^177^Lu, and ^111^In [35].

Seidl et al. depicted that the longer half-life of^ 177^Lu, as compared to ^213^Bi, prolonged the circulation time of the drug in the blood which leads to adverse effects, such as myelotoxicity. This was demonstrated in a mouse model of peritoneal carcinomatosis [36]. ^213^Bi-immunotherapy is preferable in early stage peritoneal carcinomatosis because it has good efficacy and is without toxic adverse effects. While ^177^Lu-immunotherapy is effective for late-stage disease, the adverse effects, such as myelotoxicity, make it problematic to use.

In particular, the therapeutic use of the beta-emitting radionuclide ^177^Lu has shown better results in bronchial neuroendocrine and gastroenteropancreatic tumors when compared with other therapies available [37]. For the past many years, somatostatin analogs have been the mainstay of treatment for well-differentiated tumors but not many options were available for the advanced disease [38]. PRRT with radiolabeled somatostatin has shown better outcomes in metastatic GEP-NETs [39]. Everolimus, a mammalian target of rapamycin (mTOR) inhibitor has shown good results in early as well as advanced tumors [40]. The use of everolimus in patients pretreated with ^177^Lu-octreotate radionuclide therapy is a good option in terms of safety [41].

Fluorouracil (5-FU) is routinely used to treat many malignancies [42]. The doses used caused significant toxicity. In the radiopharmaceutical technique, this molecule can be tagged with a beta-emitting radionuclide, such as ^177^Lu, and used for therapeutic purposes. This decreases the dose of 5-FU the patient is exposed to and can lead to less severe toxic adverse effects. A standardized methodology for radiolabelling of ^177^Lu-5-FU with high efficiency and stability has been developed. The biodistribution and pharmacokinetics of the radiopharmaceutical were studied in a mouse model. No cytotoxic effects were observed in vivo and very low nephrotoxicity was expected because of the short, effective half-life. This drug has the potential to be useful for the therapy of many malignancies [43].

Recent randomized trials have shown that ^177^Lu-Dotatate has very good response rates as compared to high-dose octreotide in patients with mid-gut NETs [44]. Data has shown that the effects of ^177^Lu-Dotatate are not limited to midgut NETs. Further studies are needed to compare it to everolimus, liver-directed therapies, and other systemic options. The choice of which treatment to use can depend on factors, such as the location of metastases, primary site, and level of SSTR expression. ^177^Lu-Dotatate has been recently approved for treatment of SSTR-positive advanced GEP-NETs. The NETTER-1 study (A Study Comparing Treatment With ^177^Lu-DOTA0-Tyr3-Octreotate to Octreotide LAR in Patients With Inoperable, Progressive, Somatostatin Receptor Positive Midgut Carcinoid Tumours, NCT01578239) demonstrated a 79% reduction in the risk of progression or death compared to high-dose octreotide. Adverse effects associated with ^177^Lu-Dotatate have been discussed previously. A regimen of ^177^Lu-Dotatate consists of four cycles of the drug over eight weeks. Retreatment in patients has also been shown to be beneficial [45].

In another study, ^177^Lu was combined with low-dose capecitabine chemotherapy, but the efficacy in comparison to ^177^Lu alone was not well-established [46]. Recently, a comparison was done to assess the efficacy of PRRT in using ^90^Y alone versus alternating cycles of ^90^Y and ^177^Lu. The combined therapy yielded better results than using a single agent [47]. Recent use of nanocarriers in experimental models to transport these PRRT drugs like ^177^Lu has shown decreased renal retention which can help in reducing the nephrotoxicity associated with these therapies [48].

Neuroendocrine tumor therapy with lutetium-177-octreotate and everolimus (NETTLE) study

When radiosensitizing chemotherapeutic agents are combined with PRRT, there has been a significant improvement in the efficacy of the therapeutic regimen with a modest increase in overall toxicity. To conclude, the use of everolimus in combination to PRRT has shown promising results in the efficacy of the treatment of low-grade NETs. The main side effects, when used in combination, are related to hematology, such as neutropenia and thrombocytopenia [49].

## Conclusions

Lutetium, in combination with somatostatin analogs, has proven efficacy to treat gastroenteropancreatic neuroendocrine tumors in candidates with normal renal function. This therapy decreases tumor size and improves symptoms, as well as the quality of life.
